# Supplementary material for: A moonlighting role for LysM peptidoglycan binding domains underpins Enterococcus faecalis daughter cell separation
Source: Commun Biol. 2023 Apr 18;6:428. doi: 10.1038/s42003-023-04808-z (PMC10113225; doi:10.1038/s42003-023-04808-z)
Supplement: Supplementary file 2 — Supplementary Information [file 42003_2023_4808_MOESM2_ESM.pdf]

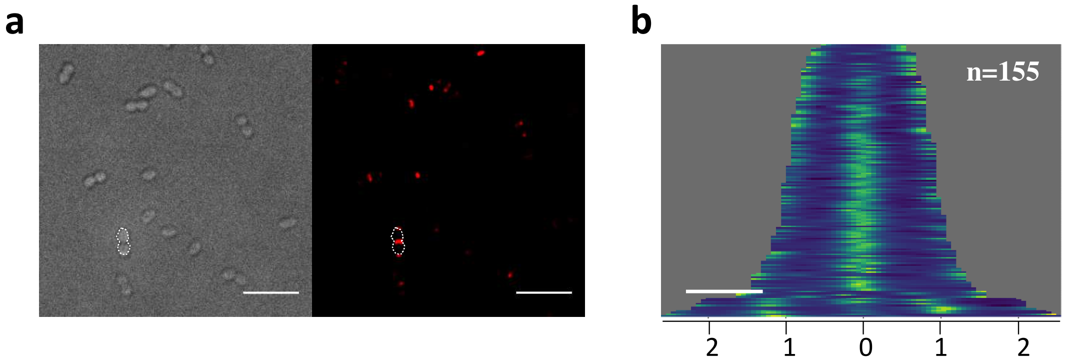

Supplementary Figure 1. Septal localization of AtlA using immunofluorescence. a. Bright-field and fluorescent images of *E. faecalis* JH2-2 probed with anti-AtlA serum and detected with anti-rabbit IgG antibodies conjugated with AlexaFluor647. Scale bars are 5 µm. **b.** Population demograph showing immunolabeled AtlA distribution in cells (n=155). Normalized fluorescent intensity of the immunolabled AtlA was quantified for each cell and the resulting heat maps of fluorescence were arranged according to cell length and stacked to generate the demographs. Scale bar in **b** is 1µm.

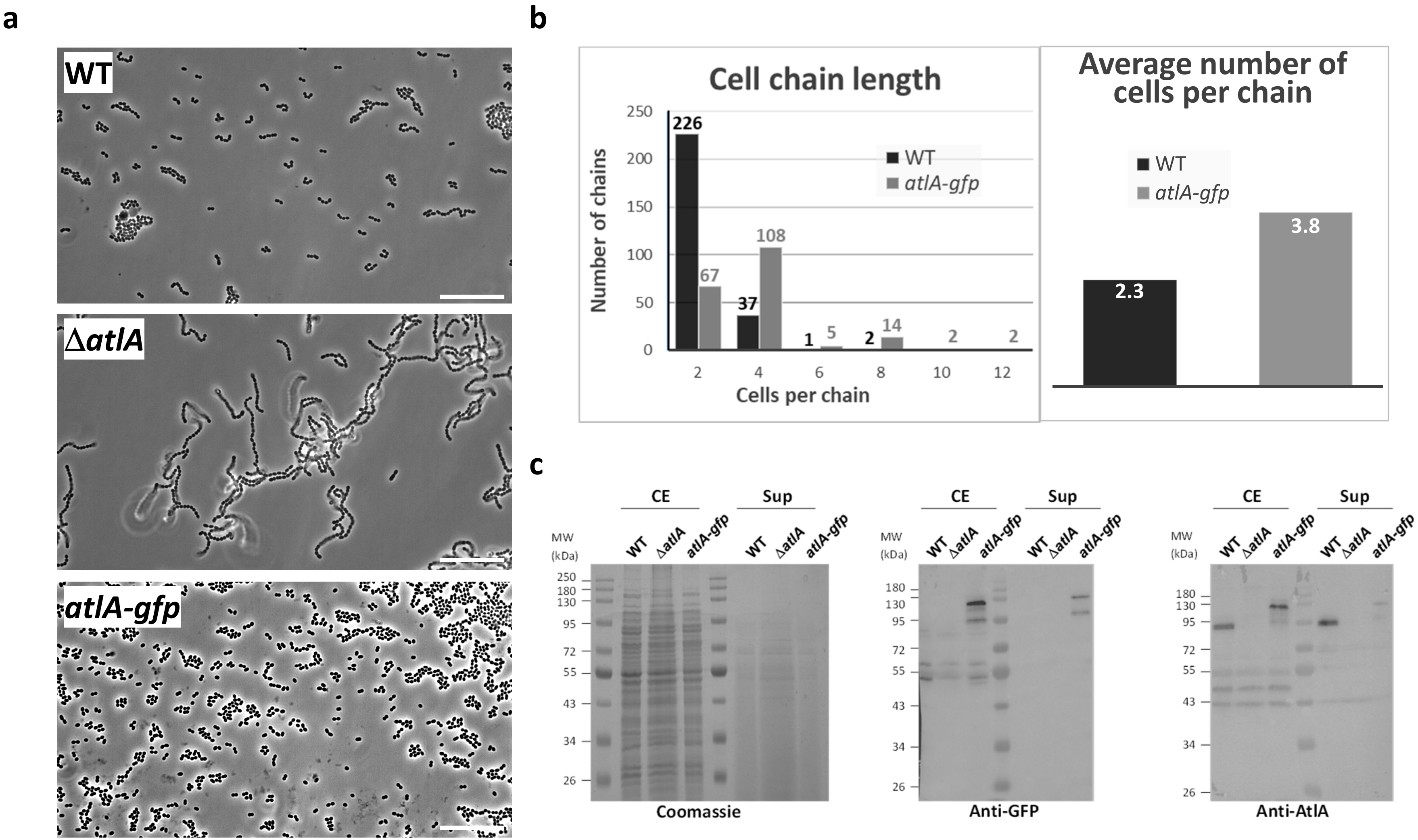

Supplementary Figure 2. Phenotypic analysis of the *atla-gfp* strain. a. Phase contrast micrographs showing wild-type JH2-2 (WT), Δ*atlA* mutant and *atlA-gfp* cells. Bar is 25µm. **b.** Cell chain length distribution in the WT and *atlA-gfp* strains. **c.** Coomassie stained SDS-PAGE of protein samples corresponding to cell lysates (CE) and culture supernatants (Sup) of WT, Δ*atlA* and *atlA-gfp* strains. Samples were transferred onto nitrocellulose and probed using anti-GFP (middle panel) and anti-AtlA antibodies (right panel).

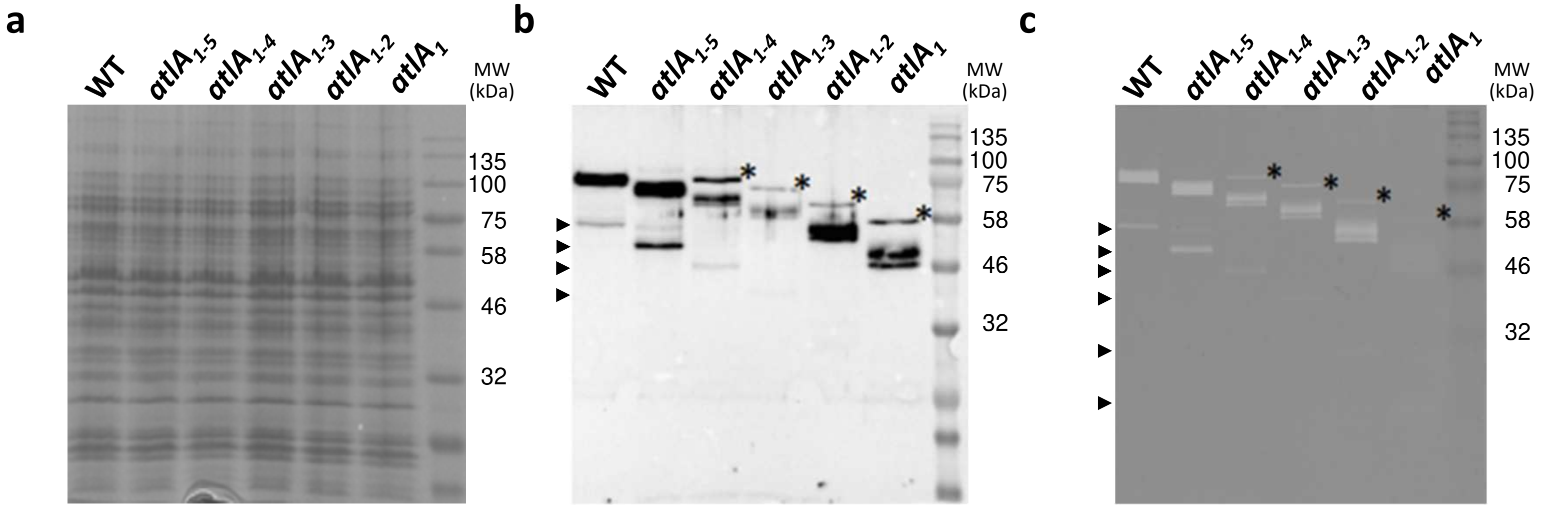

Supplementary Figure 3. Enzymatic activity of AtlA variants with truncated LysM domains. a. Coomassie-stained SDS-PAGE corresponding to whole culture extracts (cells + supernatants); JH2-2 (WT), *atlA*$_{1-5}$, *atlA*$_{1-4}$, *atlA*$_{1-3}$, *atlA*$_{1-2}$ and *atlA*$_{1}$. **b.** Immunoblotting analysis using anti-AtlA antibodies. **c**. Zymogram detection of AtlA peptidoglycan hydrolytic activity using *M. luteus* autoclaved cells as a substrate. Bands corresponding to unprocessed AtlA are indicated with asterisks. Arrows indicate proteolytic cleavage products.

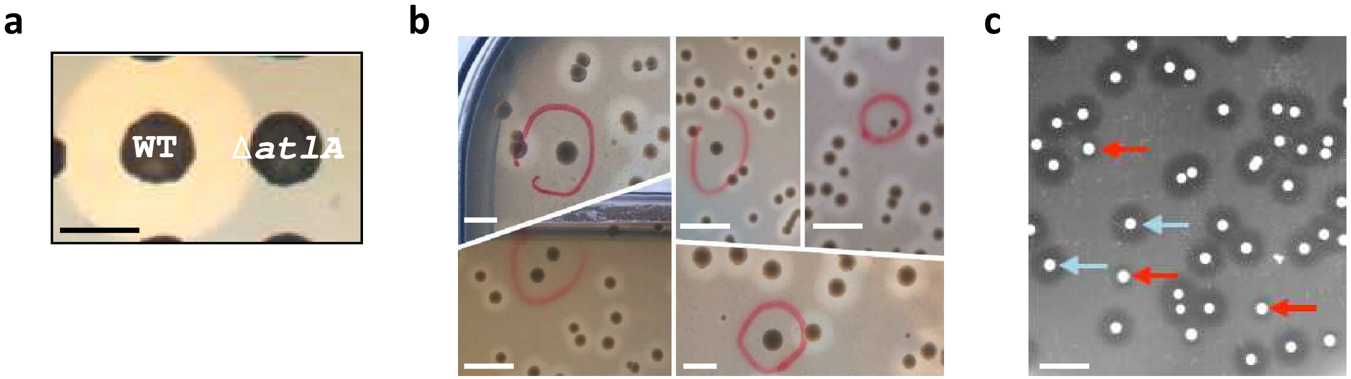

Supplementary Figure 4. Screening of transposons mutants on agar plate containing *M. luteus* autoclaved cells. **a.** Wild-type *E. faecalis* JH2-2 (WT) forms colonies surrounded by a halo corresponding to the hydrolysis of *M. luteus* peptidoglycan. The *atlA* mutant is unable to digest this substrate, indicating that this plate assay specifically detects AtlA activity. Activity was detected after 48h growth at 37°C. **b.** Several transposon mutants showing no hydrolytic activity at their cell surface (circled in red) were detected and re-isolated for further analysis. **c.** Phenotypic comparison of the wild-type and D*admA* mutant on *M. luteus* plates. This screen was used to identify mutants harbouring an in-frame deletion. The parental colonies and mutant candidates are indicated by blue and red arrows, respectively. The D*admA* mutant presents a very faint hydrolytic activity. Bar in **a**, **b** and **c** is 5mm.

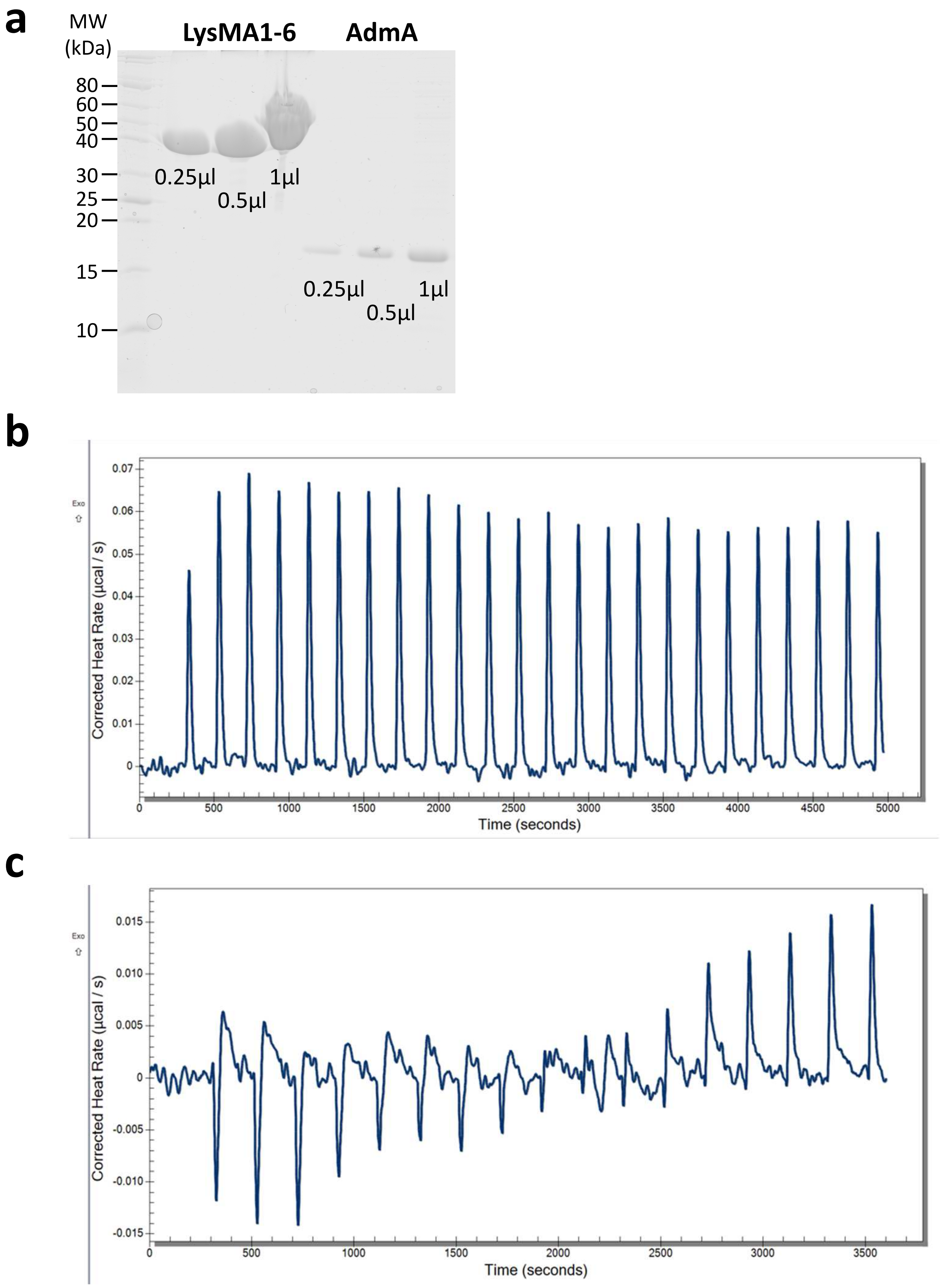

Supplementary Figure 5. Isothermal microcalorimetry profile of a titration of a 625µM LysM solution into a 67.5µM AdmA solution. a. SDS-PAGE analysis of LysM and AdmA purified recombinant proteins used for ITC experiments. Different volumes have been loaded on the gel. **b**. the area of each trough corresponds to the heat generated after the addition of 2µl of purified LysMA1-6 (625µM) to 182µl of AdmA protein solution (67.5µM). **c**. Control injections of 2µl of LysM into a buffer solution. Both proteins were purified using IMAC and gel filtration in 50 mM Tris pH 8.5, 150 mM NaCl containing 0.05% (v/v) tween-20.

a

	Split luciferase fusion	
	Small Fgt	Large fgt
1	Full length luciferase	
2	AtlA	AdmA
3	AtlA	-
4	-	AdmA
5	AtlAΔLysM	AdmA
6	AtlAΔLysM	-
7	LysM	AdmA
8	LysM	-

c

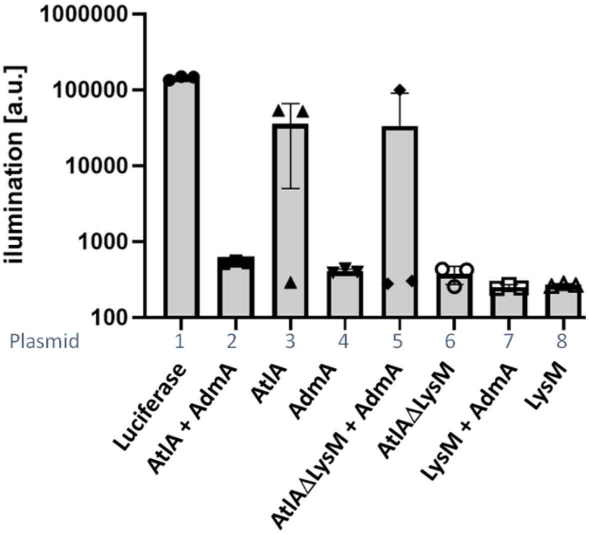

b

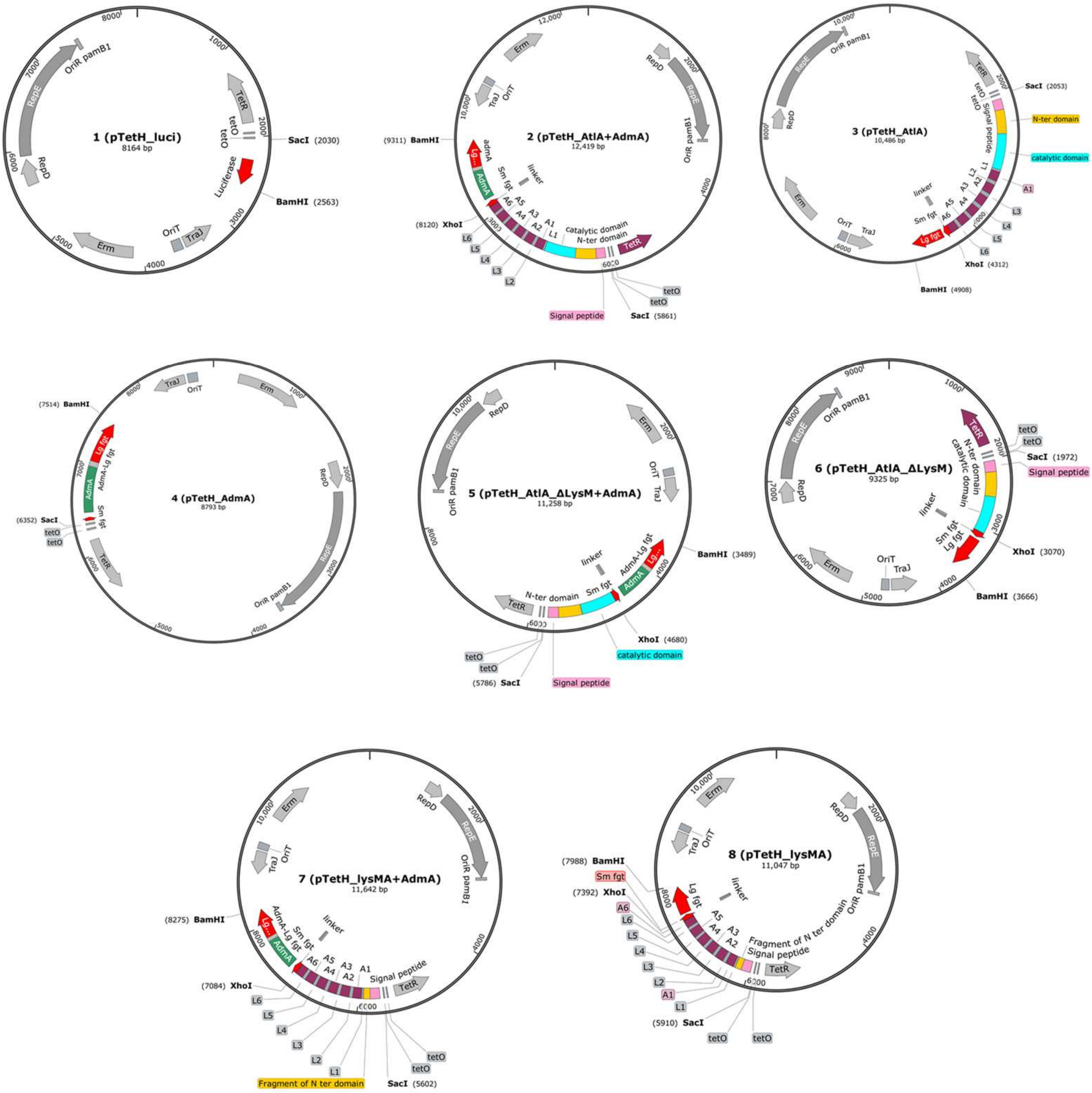

Supplementary Figure 6. Split luciferase assays. a. Description of the fusions encoded by all plasmids used for split luciferase assays. **b.** Plasmid maps describing the luciferase fusions and controls used. **c.** Luciferase assays; all plasmids were transformed in *E. faecalis* JH2-2 and luciferase activity was tested as described previously (doi: 10.1021/acssynbio.6b00104). Error bars are standard error of mean.

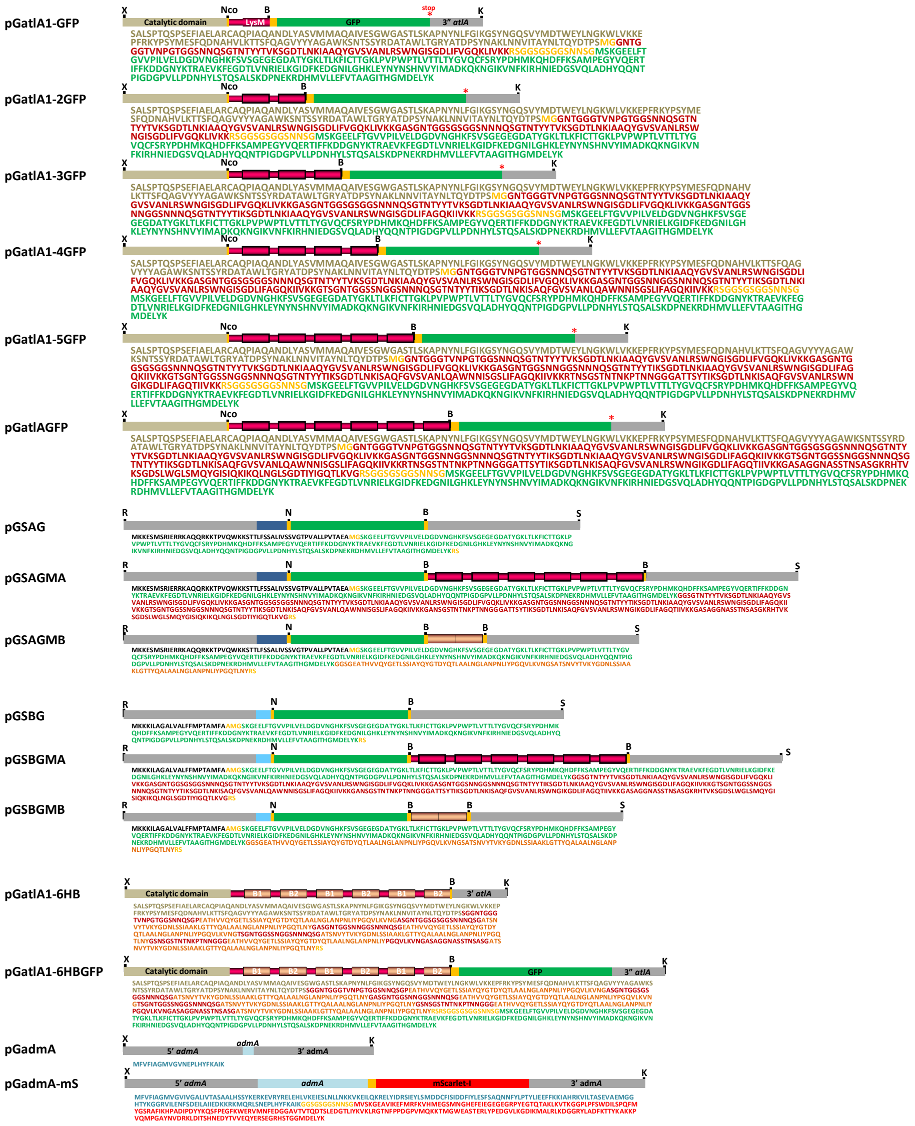

Supplementary Figure 7. Schematic representation of proteins encoded by gene replacement plasmids and corresponding sequences.

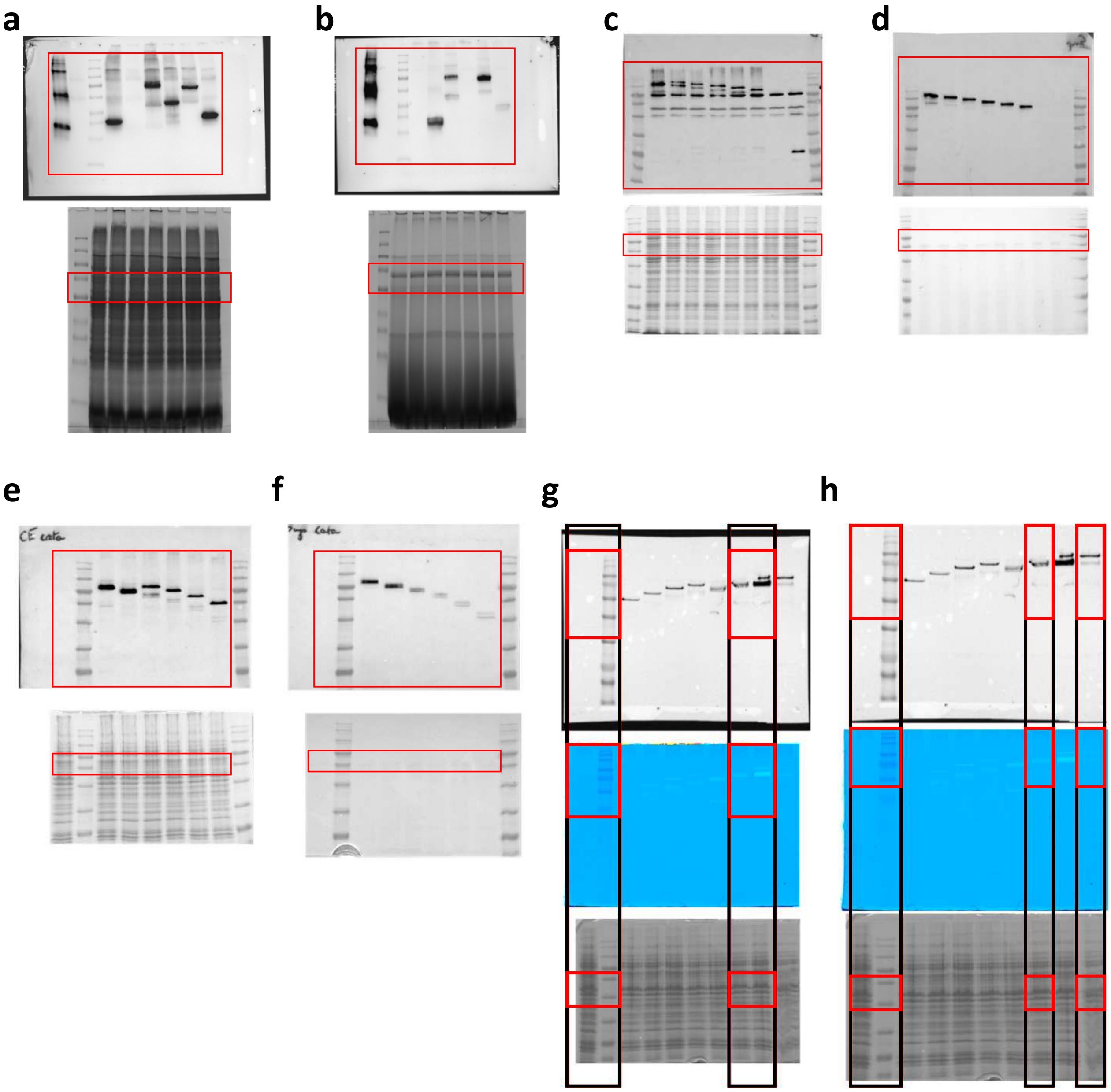

Supplementary Figure 8. Uncropped and unedited blots and Coomassie gels. a, Figure 1e; **b,** Figure 1f; **c**, Figure 2b; **d.**, Figure 2c; **e**, Figure 3b; **f**, Figure 3c; **g**, Figure 4c; **h**, Figure 5b

Supplementary Table 1. Description of all transposon mutants showing a reduced AtlA activity on *M. luteus* plates.

Gene ID[a]	Number of mutants	Insertion site (nt position)	Function
EF_0018	2	700<br>1239	s[54] interaction domain-containing protein
EF_0020 [b]	1	809	PTS system mannose transporter IIAB
EF_0773	1	195	*admA*
EF_0799	15	362, 613, 675, 680, 478, 787, 728, 861, 980, 985, 1249, 1290, 1292, 1963	*atlA*
EF_0954	2	325<br>508	Sugar-binding transcriptional regulator (LacI family)
EF_2674	1	677	Oligoendopeptidase F
EF_3156	1	104	GntR transcriptional regulator

[a] Gene ID relates to the annotated *E. faecalis* V583 genome.

[b] These mutants present a bigger halo, indicating an increased AtlA activity.

Supplementary Table 2. Bacterial strains, plasmids and oligonucleotides used in this study.

Strains, plasmids or oligonucleotides	Relevant properties or genotype[a]	Source or reference
**Strains**		
Enterococcus faecalis		
OG1RF	Plasmid-free, virulent laboratory strain isolated from the oral cavity	Dunny *et al.*, 1978
JH2-2	Plasmid-free laboratory strain	Jacob & Hobbs, 1974
Δ*atlA*	JH2-2 mutant harboring an in-frame deletion of *atlA*	Mesnage et al., 2008
$s_{A}g$	JH2-2 producing the GFP secreted via the signal peptide of AtlA	This work
$s_{A}gm_{A}$	JH2-2 producing a GFP-$LysM_A$ fusion secreted via the signal peptide of AtlA	This work
$s_{A}gm_{B}$	JH2-2 producing a GFP-$LysM_B$ fusion secreted via the signal peptide of AtlA	This work
$s_{B}g$	JH2-2 producing the GFP secreted via the signal peptide of AtlB	This work
$s_{B}gm_{A}$	JH2-2 producing a GFP-$LysM_A$ fusion secreted via the signal peptide of AtlB	This work
$s_{B}gm_{B}$	JH2-2 producing a GFP-$LysM_B$ fusion secreted via the signal peptide of AtlB	This work
$atlA_{1}$	JH2-2 producing AtlA with a LysM domain lacking the last 5 LysM repeats	Salamaga et al., 2017
$atlA_{1\text{-}2}$	JH2-2 producing AtlA with a LysM domain lacking the last 4 LysM repeats	Salamaga et al., 2017
$atlA_{1\text{-}3}$	JH2-2 producing AtlA with a LysM domain lacking the last 3 LysM repeats	Salamaga et al., 2017
$atlA_{1\text{-}4}$	JH2-2 producing AtlA with a LysM domain lacking the last 2 LysM repeats	Salamaga et al., 2017
$atlA_{1\text{-}5}$	JH2-2 producing AtlA with a LysM domain lacking the last LysM repeat	Salamaga et al., 2017
$atlA_{1}$-*gfp*	$atlA_{1}$ producing an AtlA variant fused to the GFP	This work
$atlA_{1\text{-}2}$-*gfp*	$atlA_{1\text{-}2}$ producing an AtlA variant fused to the GFP	This work
$atlA_{1\text{-}3}$-*gfp*	$atlA_{1\text{-}3}$ producing an AtlA variant fused to the GFP	This work
$atlA_{1\text{-}4}$-*gfp*	$atlA_{1\text{-}4}$ producing an AtlA variant fused to the GFP	This work
$atlA_{1\text{-}5}$-*gfp*	$atlA_{1\text{-}5}$ producing an AtlA variant fused to the GFP	This work
atlA-gfp	JH2-2 producing the AtlA-GFP translational fusion	This work
$atlA_{1\text{-}6HB}$	JH2-2 *producing* AtlA with a LysM domain made of 3 sets of LysM repeats from AtlB	This work
$atlA_{1\text{-}6HB}$-*gfp*	$atlA_{1}$ producing an AtlA variant fused to the GFP	This work
Δ*admA*	JH2-2 mutant harboring an in-frame deletion of *admA*	This work
admA-mS	JH2-2 producing the AdmA-mScarlet-I translational fusion	This work
atlA-gfp admA-mS	JH2-2 producing the AdmA-mScarlet-I and AtlA-GFP translational fusions	This work
$atlA_{E212Q}$	JH2-2 producing AtlA allele with a single amino acid substitution (E212Q) abolishing AtlA activity	Zamboni *et al.*, 2022
Escherichia coli		
NEB5α	Host for plasmid propagation	NEB
TG1(*RepA*)	TG1 derivative harboring *RepA* for pGhost propagation at 37°C	P. Serror
**Plasmids**		
pGhost9	Thermosensitive plasmid for targeted gene replacement in *E. faecalis* (Erm^R)	Maguin et al., 1992
pZXL5	Thermosensitive plasmid for transposon mutagenesis (Gm^R)	Zhang et al. 2012
pTetH	pAT18 derivative for tetracycline-inducible expression in *E. faecalis*	Smith et al., 2019
pGSAG	pGhost9 derivative used to generate strains secreting GFP via the AtlA signal peptide	This work
pGSAGMA	pGhost9 derivative used to generate strains secreting GFP via the AtlA signal peptide	This work
pGSAGMB	pGhost9 derivative used to generate strains secreting GFP via the AtlA signal peptide	This work
pGSBG	pGhost9 derivative used to generate strains secreting GFP via the AtlB signal peptide	This work
pGSBGMA	pGhost9 derivative used to generate strains secreting GFP via the AtlB signal peptide	This work
pGSBGMB	pGhost9 derivative used to generate strains secreting GFP via the AtlB signal peptide	This work
pGatlA-GFP	pGhost9 derivative used to generate strain *atlA-gfp*	This work
pGatlA1-5-GFP	pGhost9 derivative used to generate strain $atlA_{1\text{-}5}$-*gfp*	This work
pGatlA1-4-GFP	pGhost9 derivative used to generate strain $atlA_{1\text{-}4}$-*gfp*	This work
pGatlA1-3-GFP	pGhost9 derivative used to generate strain $atlA_{1\text{-}3}$-*gfp*	This work
pGatlA1-2-GFP	pGhost9 derivative used to generate strain $atlA_{1\text{-}2}$-*gfp*	This work
pGatlA1-GFP	pGhost9 derivative used to generate strain $atlA_{1}$-*gfp*	This work
pGatlA1-6HB	pGhost9 derivative used to generate strain $atlA_{1\text{-}6HB}$	This work

pGatlA1-6HB-GFP	pGhost9 derivative used to generate strain *atlA*$_{1\text{-6HB}}$-*gfp*	This work
pGadmA	pGhost9 derivative carrying an in-frame deletion of *admA*	This work
pGadmA-mS	pGhost9 derivative used to construct *admA-mScarlet*	This work
pTeTH-admA	pTETH encoding full length AdmA for complementation	This work

Oligonucleotides

AdmA_H11	AAACTCGAGTCTAAAATGTGACGAGCAGCAATTTC
AdmA_H12	CGCTCCACTTCCTGAACCACCGACGATTACACCGACCATTC
AdmA_H21	GGTTCAGGAAGTGGAGCGATTAAATAAAAAAAGAACCAGCGT
AdmA_H22	TATGAATTCGTTTAGCCCGTTTATTTCAACAATATTTGAC
AdmA_G_Fw	ACAGATCTGAGCTCAAGGAGGAGACTGACCATGGTGTTTGTTTTTATCGCCGGAATGGTCG
AdmA_G_Rev	CTTTAGTGATGATGGTGATGGTGATGGTGGGATCCTTTAATCGCTTTAAAATAATGAAGTG
atlA_G_Fw	ATCAAGCTTATCGATACCGTCGACCTCGAGTTTTTCAGCGTTATCACCGACGCAAAGT
atlA1-6_G_Rev	TTTTAAAGTTTGACCAATATAAATTGTATCCCCGC
6L_GFP_G_Fw	GATACAATTTATATTGGTCAAACTTTAAAAGTTGGTGGATCCGGAGGATCAGGTAGT
L-GFP_G_Rev	TACGACTCACTATAGGGCGAATTGGGTACCACCTAAATTCGTCAAAGAAACGGTCAG
atlA1-5_G_Rev	TTTTTTCACGATGATTGTTTGACCAGCAAAAATT
5L-GFP_G_Fw	TTTGCTGGTCAAACAATCATCGTGAAAAAAGGATCCGGAGGATCAGGTAGT
atlA1-4_G_Rev	TTTTTTCACGATAATCTTTTGACCAGCAAAAATCAAACTACCGC
4L-GFP_G_Fw	TTTGCTGGTCAAAAGATTATCGTGAAAAAAGGATCCGGAGGATCAGGTAGT
atlA1-3_G_Rev	TTTTTTCACAATAATTTTTTGACCAGCGAAGA
3L-GFP_G_Fw	TTCGCTGGTCAAAAAATTATTGTGAAAAAAGGATCCGGAGGATCAGGTAGT
atlA1-2_G_Rev	TTTTTTCACGATGAGTTTTTGACCAACGAAAATTAAATCGCCAGAGATGCCAT
2L-GFP_G_Fw	TTCGTTGGTCAAAAACTCATCGTGAAAAAAGGATCCGGAGGATCAGGTAGT
atlA1_G_Rev	TTTTTTCACGATAAGTTTTTGACCAAC
1L-GFP_G_Fw	TTCGTTGGTCAAAAACTTATCGTGAAAAAAGGATCCGGAGGATCAGGTAGT
SAG_G_Fw	CGTTACTACCAGTGACTGCTGAGGCCATGGGTAGTAAAGGAGAAGAACTTTTCACTGG
SAG_G_Rev	GCTATTTTTTTCAATCTTAAATTAAGATCTTTTGTATAGTTCATCCATGCCATGTGTA
SAGMA_Bgl_Fw	CATGGCATGGATGAACTATACAAAAGATCTGGAACGAACACGTACTATACTGTAAAATCAGGA
SAGMA_Bgl_Rev	TTAAGATCTACCAACTTTTAAAGTTTGACCAATATAAATTGTATCCCC
SAGMB_Bgl_Fw	AAGATCTGGAGAAGCAACACACGTGGTTCAATATG
SAGMB_Bgl_Rev	TAAGATCTATAATTCAATGTTTGGCCAGGATAAATCAAATTAGGATTTGA
Mar_up	GCTAATGCAGTTATTTTAGACCCTC
Mar_dn	GGTAGCTTTTTAAATATGGCGCTTC
T7	TAATACGACTCACTATAGG

Amp^{R}, resistant to ampicillin; Erm^{R}, resistant to erythromycin

Supplementary Table 3. Plasmid construction strategy and sequences of synthetic gene fragment used.

Plasmid	Derivative of	Cloning strategy	Primers/ Gene synthesis
pGSAG	pGHH0799 (Mesnage et al., 2008)	PCR from chromosomal DNA cloned into pGHH0799 cut with NcoI + BglII by Gibson assembly	SAG_G_Fw and SAG_G_Rev
pGSAGMA	pSAG	PCR from chromosomal DNA, digestion with BglII and cloning into pSAG cut with BglII and dephosphorylated	SAGMA_Bgl_Fw SAGMA_Bgl_Rev
pGSAGMB	pSAG	PCR from chromosomal DNA, digestion with BglII and cloning into pSAG cut with BglII and dephosphorylated	SAGMB_Bgl_Fw SAGMB_Bgl_Rev
pGSBG	pGHH0799 (Mesnage et al., 2008)	**Restriction cloning of a synthetic gene fragment cut with EcoRI+NcoI cloned in pGost9**	GS1
pGSBGMA	pSBG	PCR from chromosomal DNA, digestion with BglII and cloning into pSAG cut with BglII and dephosphorylated	SAGMA_Bgl_Fw SAGMA_Bgl_Rev
pGSBGMB	pSBG	PCR from chromosomal DNA, digestion with BglII and cloning into pSAG cut with BglII and dephosphorylated	SAGMB_Bgl_Fw SAGMB_Bgl_Rev
pGatlA-GFP	pGhost	PCR from chromosomal DNA (*atlA*) and Gene synthesis (linker-*gfp*-3'*atlA*) followed by 3 fragment Gibson assembly in pGhost9 cut with XhoI + KpnI	atlA_G_Fw, atlA1-6_G_Rev 6L_GFP_G_Fw, L-GFP_G_Rev GS2
pGatlA1-5GF	pGhost	PCR from chromosomal DNA (atlA1-A5) and Gene synthesis (linker-*gfp*-3'*atlA*) followed by 3 fragment Gibson assembly in pGhost9 cut with XhoI + KpnI	atlA_G_Fw, atlA1-5_G_Rev 5L_GFP_G_Fw, L-GFP_G_Rev GS2
pGatlA1-4GF	pGhost	PCR from chromosomal DNA (atlA1-A4) and Gene synthesis (linker-*gfp*-3'*atlA*) followed by 3 fragment Gibson assembly in pGhost9 cut with XhoI + KpnI	atlA_G_Fw, atlA1-4_G_Rev 4L_GFP_G_Fw, L-GFP_G_Rev GS2
pGatlA1-3GF	pGhost	PCR from chromosomal DNA (atlA1-A3) and Gene synthesis (linker-*gfp*-3'*atlA*) followed by 3 fragment Gibson assembly in pGhost9 cut with XhoI + KpnI	atlA_G_Fw, atlA1-3_G_Rev 3L_GFP_G_Fw, L-GFP_G_Rev GS2
pGatlA1-2GF	pGhost	PCR from chromosomal DNA (atlA1-A2) and Gene synthesis (linker-*gfp*-3'*atlA*) followed by 3 fragment Gibson assembly in pGhost9 cut with XhoI + KpnI	atlA_G_Fw, atlA1-2_G_Rev 2L_GFP_G_Fw, L-GFP_G_Rev GS2
pGatlA1-GFP	pGhost	PCR from chromosomal DNA (atlA1) and Gene synthesis (linker-*gfp*-3'*atlA*) followed by 3 fragment Gibson assembly in pGhost9 cut with XhoI + KpnI	atlA_G_Fw, atlA1_G_Rev 1L_GFP_G_Fw, L-GFP_G_Rev GS2
pGatlA-6HB	pGhost9	Restriction cloning of a synthetic gene fragment cut with XhoI+EcoRI cloned in pGost9	GSZ

pGatlA-6HB-G	pGatlA1-6HB	Restriction cloning of a synthetic gene fragment cut with BglII+XhoI cloned in pGatlA-6HB	GSO
pGadmA	PGhost	Overlap extension PCR, restriction cloning in pGhost9 cut with XhoI+EcoRI cloned in pGhost9	AdmA_H11, AdmA_H12, AdmA_H21, AdmA_H22
pGadmA-mS	pGhost9	Restriction cloning of a synthetic gene fragment cut with XhoI-EcoRI gene synthesis in pGhost9	GS5
pTetH-admA	pTetH	PCR, Gibson assembly in pTetH cut with NcoI + BamHI	AdmA_G_Fw, AdmA_G_Rev

Gene synthesis	Sequence
GS1 (5'atlA-SB)	GAATTCTTCGTTCCGTCAATTCAATGCCTTGTTCTTTCAAAATTTCTTCTGCGATAACCCGTGGTTTCCCCAATGATTTTGCCACTTGTTCTTCTGTTTCGCCTTCCGCAACACG<br>AGCATCAAATAACGCCTTGTATTTCGCAAGAATAATAGCTTGATCTTTTGGATTTAATGCTTTTAAATAAATTTTCAGCTCAATAATAAAATGTTCTTTATTCATTCCGCTCCCT<br>CTTTCTACACGATTCATTTCTCATCTTAAACTATAATTTCTTTTTGGGCGAATAACAAGAGGAATTAAAAAAATCAGACTAAGGTAAAGAAGCGTCTACAACTAAAGTCGCAGTT<br>TTTTTCCGAGTAGTAACAAGAGAGTATATCCGTGTTAAAAAACATTACTTCTTTACAATCTAGTTACAGTAATATGAAATTTTGTTGAAGTGTTTTTAATTCGCCAGAAAAAAAG<br>GTATACTTGTTATAATAACAAAACTAAAAAATTACATTTACATATAAGTTAAAAAAGGAAAGTTGGGGACGTATCAATGAAGAAGAAAATCTTAGCAGGAGCGCTTGTCGCTCTG<br>TTTTTTATGCCTACAGCTATGTTTGCCGCCATG
GS2 (L-GFP-3'atlA)	GGATCCGGAGGATCAGGTAGTGGAGGAAGCAATAATAGTGGTATGAGTAAAGGAGAAGAACTTTTCACTGGAGTTGTCCCAATTCTTGTTGAATTAGATGGTGATGTTAATGGGC<br>ACAAATTTTCTGTCAGTGGAGAGGGTGAAGGTGATGCAACATACGGAAAACTTACCCTTAAATTTATTTGCACTACTGGAAAACTACCTGTTCCGTGGCCAACACTTGTCACTAC<br>TTTGACTTATGGTGTTCAATGCTTTTCAAGATACCCAGATCATATGAAACAGCATGACTTTTTCAAGAGTGCCATGCCCGAAGGTTATGTACAGGAAAGAACTATATTTTTCAAA<br>GATGACGGGAACTACAAGACACGTGCTGAAGTCAAGTTTGAAGGTGATACCCTTGTTAATAGAATCGAGTTAAAAGGTATTGATTTTAAAGAAGATGGAAACATTCTTGGACACA<br>AATTGGAATACAACTATAACTCACACAATGTATACATCATGGCAGACAAACAAAAGAATGGAATCAAAGTTAACTTCAAAATTAGACACAACATTGAAGATGGAAGCGTTCAACT<br>AGCAGACCATTATCAACAAAATACTCCAATTGGCGATGGCCCTGTCCTTTTACCAGACAACCATTACCTGTCCACACAATCTGCCCTTTCGAAAGATCCCAACGAAAAGAGAGAT<br>CACATGGTCCTTCTTGAGTTTGTAACAGCTGCTGGGATTACACATGGCATGGATGAACTATACAAATAGTTGGTTAATTTAAGATTGAAAAAAATAGCTATCTTTGGTAACATGA<br>GTCTTATAATAGAAAAAAACAATGAGAAGGAATAGTAGAAAGAACCATGTGATAGAGAGCGTATGGCTGGTGGAAATACGTACAGAAGCTTTTGAACTCGCCTTTAAGTTACTTT<br>TTTGAACAAACCAAGTAGGAAAAGTCGGTGACGATCGTTAAGACGTTTGAGGTTAAGGTGACGGTGACTGACCGTTTCTTTGACGAATTTAGGTGGTACCCAATTCGCCCTATAG<br>TGAGTCGTA
GS3 (atlA-6HB)	GAATTCTCAGCGTTATCACCGACGCAAAGTCCTTCAGAATTTATTGCCGAGTTAGCTCGTTGTGCACAACCTATTGCGCAAGCCAATGATTTATATGCATCAGTGATGATGGCTC<br>AAGCAATCGTTGAAAGTGGTTGGGGAGCAAGTACGCTATCTAAGGCACCAAACTATAACTTATTTGGGATTAAAGGCAGCTACAATGGACAATCTGTCTATATGGATACATGGGA<br>ATATTTAAACGGCAAATGGTTAGTGAAAAAAGAACCTTTCCGTAAATATCCTTCTTACATGGAATCATTCCAAGATAATGCGCACGTGCTAAAAACAACTTCTTTCCAAGCGGGC<br>GTTTACTATTATGCTGGGGCTTGGAAAAGCAATACAAGCTCGTACCGCGATGCAACTGCTTGGTTAACAGGTCGTTATGCGACAGATCCTAGCTACAATGCTAAATTAAATAATG<br>TCATTACCGCATATAACTTAACTCAATATGATACACCATCTTCTGGTGGAAATACTGGGGGCGGAACAGTTAATCCAGGAACAGGCGGCTCGAACAATCAATCAGGACCAGAAGC<br>GACTCATGTGGTACAATACGGAGAAACATTATCAAGTATTGCTTATCAATATGGAACAGACTATCAAACGTTGGCGGCATTAAATGGATTGGCTAATCCAAATCTTATTTATCCT<br>GGTCAAGTTTTGAAAGTCAATGGTGCTTCAGGTAACACTGGTGGCTCAGGCAGCGGTGGTTCTAACAATAATCAATCAGGAGCAACAAGTAATGTCTACACGGTTAAATACGGCG<br>ATAATTTATCTAGTATTGCAGCAAAACTTGGCACTACTTATCAAGCTTTAGCTGCATTAAACGGATTAGCAAATCCTAACTTGATTTATCCAGGTCAAACATTGAATTATGGTGC<br>TTCAGGTAACACTGGTGGCTCAAACAACGGTGGCTCTAACAATAATCAATCAGGAGAAGCGACTCATGTGGTACAATACGGAGAAACATTATCAAGTATTGCTTATCAATATGGA<br>ACAGACTATCAAACGTTGGCGGCATTAAATGGATTGGCTAATCCAAATCTTATTTATCCTGGTCAAGTTTTGAAAGTCAATGGTACTTCAGGTAACACCGGTGGCTCAAGCAATG<br>GTGGTTCTAACAATAATCAATCAGGAGCAACAAGTAATGTCTACACGGTTAAATACGGCGATAATTTATCTAGTATTGCAGCAAAACTTGGCACTACTTATCAAGCTTTAGCTGC<br>ATTAAACGGATTAGCAAATCCTAACTTGATTTATCCAGGTCAAACATTGAATTATGGATCCAACTCAGGTTCAACGAATACGAACAAGCCTACGAATAATGGTGGCGGTGAAGCG<br>ACTCATGTGGTACAATACGGAGAAACATTATCAAGTATTGCTTATCAATATGGAACAGACTATCAAACGTTGGCGGCATTAAATGGATTGGCTAATCCAAATCTTATTTATCCTG<br>GTCAAGTTTTGAAAGTCAATGGCGCTTCTGCAGGTGGCAATGCTTCTTCAACAAATAGTGCATCAGGCGCAACAAGTAATGTCTACACGGTTAAATACGGCGATAATTTATCTAG<br>TATTGCAGCAAAACTTGGCACTACTTATCAAGCTTTAGCTGCATTAAACGGATTAGCAAATCCTAACTTGATTTATCCAGGTCAAACATTGAATTATAGATCTTGATAATTTAAG<br>ATTGAAAAAAATAGCTATCTTTGGTAACATGAGTTTTATAATAGAAAAAAACAATGAGAAGGAATAGTAGAAAGAACCATGTGATAGAGAGCGTATGGCTGGTGGAAATACGTAC<br>AGAAGCTTTTGAACTCGCCTTTAAGTTACTTTTTTGAACAAACCAAGTAGGAAAAGTCGGTGACGATCGTTAAGACGTTTGAGGTTAAGGTGACGGTGACTGACCGTTTCTTTGA<br>CGAATTTAGGTCTCGAG
GS4 (atlA-6HB-GFP	AGATCTGGAGGATCAGGTAGTGGAGGAAGCAATAATAGTGGTATGAGTAAAGGAGAAGAACTTTTCACTGGAGTTGTCCCAATTCTTGTTGAATTAGATGGTGATGTTAATGGGC<br>ACAAATTTTCTGTCAGTGGAGAGGGTGAAGGTGATGCAACATACGGAAAACTTACCCTTAAATTTATTTGCACTACTGGAAAACTACCTGTTCCGTGGCCAACACTTGTCACTAC<br>TTTGACTTATGGTGTTCAATGCTTTTCAAGATACCCAGATCATATGAAACAGCATGACTTTTTCAAGAGTGCCATGCCCGAAGGTTATGTACAGGAAAGAACTATATTTTTCAAA<br>GATGACGGGAACTACAAGACACGTGCTGAAGTCAAGTTTGAAGGTGATACCCTTGTTAATAGAATCGAGTTAAAAGGTATTGATTTTAAAGAAGATGGAAACATTCTTGGACACA<br>AATTGGAATACAACTATAACTCACACAATGTATACATCATGGCAGACAAACAAAAGAATGGAATCAAAGTTAACTTCAAAATTAGACACAACATTGAAGATGGAAGCGTTCAACT<br>AGCAGACCATTATCAACAAAATACTCCAATTGGCGATGGCCCTGTCCTTTTACCAGACAACCATTACCTGTCCACACAATCTGCCCTTTCGAAAGATCCCAACGAAAAGAGAGAT

	CACATGGTCCTTCTTGAGTTTGTAACAGCTGCTGGGATTACACATGGCATGGATGAACTATACAAATAGTTGGTTAATTTAAGATTGAAAAAAATAGCTATCTTTGGTAACATGA<br>GTCTTATAATAGAAAAAAACAATGAGAAGGAATAGTAGAAAGAACCATGTGATAGAGAGCGTATGGCTGGTGGAAATACGTACAGAAGCTTTTGAACTCGCCTTTAAGTTACTTT<br>TTTGAACAAACCAAGTAGGAAAAGTCGGTGACGATCGTTAAGACGTTTGAGGTTAAGGTGACGGTGACTGACCGTTTCTTTGACGAATTTAGGTGGTACCACGTTGCATTTGTAT<br>GTTCACGTCCTATAGGAATTTTTCCTATAGGACTTTTTTTTATCACTAAGTGGCTAGTTTAAGTCAGAATAGTGACAATGGCTGCAGGTGGCAAATTTTAAGAATGAAAAATTTT<br>ATATTACTAGGAGGAATAACATGAGCTACAATCACAAACTCGAG
**(AdmA-mScarlet)**	AGAATGCCTATGAAAAGTATACCTCTTTGGTCAATAAAGGTCAACGAAGTCGCTTGAAAAATTTTGTGTCATTTTTTATCCGAAACTTTTGGATTATGTTATACTAAAATGGAAAATT<br>AAGAGCGAGGGGAAAAATATGTTTGTTTTTATCGCCGGAATGGTCGGTGTAATCGTCGGTGCGCTAATAGTCACAGCAAGTGCCGCCTTACATTCATCTTACAAAGAGCGAAAAGAAG<br>TTATCGTGAACTAGAACATTTGGTTAAAGAAATTGAATCATTGAATTTATTAAATAAAAAAGTCAAAGAAATTTTACAAAAGCGTGAATTATATATAGACAGATCAATTGAATACTTA<br>TGGATGATTGTTTCATCAGCATCGATGATTTTATTTATTTGGAATCATTTTCTGCTCAAAATAATTTTTACTTACCGACGTACTTAATTGAAGAATTTTTCAAAAAAATTGCGCATCG<br>GTCATTTTGACGGCCAGCGAAGTTGCTGAAATGGGTGGTCACACCTATAAAGGCGGCCGTGTTATTTTAGAAAATTTTTCAGACGAGATTTTAGCAATTATTGAAGATAAAAAACGTA<br>GCAGCGTTTGTCTAACGAACCACTTCATTATTTTAAAGCGATTAAAGGAGGATCAGGTAGTGGAGGAAGCAATAATAGTGGTATGGTTTCAAAAGGAGAGGCAGTGATAAAGGAGTTC<br>GTTTTAAGGTGCATATGGAAGGGTCTATGAATGGTCACGAGTTCGAAATTGAGGGTGAAGGGGAGGGGAGACCTTACGAGGGCACTCAGACTGCGAAGTTGAAGGTAACGAAGGGCGG<br>TTACCTTTCAGCTGGGATATACTTTCACCTCAATTTATGTATGGGAGTCGTGCTTTTATAAAACATCCTGCGGACATCCCTGATTATTACAAGCAATCATTTCCTGAAGGCTTTAAGT<br>GAGAGTTATGAATTTTGAGGATGGCGGCGCTGTAACTGTGACCCAAGATACATCTTTAGAGGATGGTACCCTTATCTATAAGGTGAAACTTAGAGGGACGAACTTTCCGCCAGATGGA<br>TTATGCAGAAAAAAACTATGGGTTGGGAAGCCTCAACCGAGCGACTTTACCCGGAAGATGGTGTCCTTAAGGGCGACATAAAGATGGCTCTTCGATTAAAAGACGGCGGCAGATACTT<br>GATTTTAAGACAACATACAAAGCGAAGAAACCTGTCCAGATGCCAGGGGCCTATAATGTTGACAGAAAATTGGACATCACAAGCCACAACGAAGATTATACTGTTGTGGAACAGTACG<br>TAGCGAGGGCCGTCACTCTACGGGGGGAATGGACGAATTATATAAGTAAAAAAAGAACCAGCGTACCCGCTAATTTTTTGGGGTATCTGGTTCTTTTTCTTGTTGTTCCATATCGGTC<br>TGTCTTCAATTTCTTTATGTAAGAAATAAGAAATAACTGTACGGATGATCACCAAGGTTGCCAATTTCAAAATATCTTGAAAAGTTGGTTTGACAATGGATTCAATAATATCAGCAGC<br>AAAATTTCTAAACTTAACAGAATGTAGCTACCAAGGAAGTTTTTGATGAACGTATTCATTTTGGTTAAGGTAAAGCGGCTTTGATTTTGTCGTTCACTTTTAATGAAATCTTTTCCTG<br>ACAAACACCCCAAACTAAAACAATAATCGAAAAAACATTCAGTCCTAAAATACATAGCTCAAAAAGTGGGGTTAAAAGCGTCATTAACTGATGGGGCCACTCATTCATCGTATTCACT<br>CCAAAAAGCCTTTTCTTTAACTATAACGAAACGGCTGACTAACGTCAAATATTGTTGAAATAAACGGCTAAACGAATTC
