# Supplementary material for: A moonlighting role for LysM peptidoglycan binding domains underpins Enterococcus faecalis daughter cell separation
Source: Commun Biol. 2023 Apr 18;6:428. doi: 10.1038/s42003-023-04808-z (PMC10113225; doi:10.1038/s42003-023-04808-z)
Supplement: Supplementary file 3 — Description of Additional Supplementary Files [file 42003_2023_4808_MOESM3_ESM.pdf]

Description of Additional Supplementary Files

File Name: Supplementary Data 1
Description: Source data underlying Figures 1b, 4b and 5a
