# Supplementary material for: A moonlighting role for LysM peptidoglycan binding domains underpins Enterococcus faecalis daughter cell separation
Source: Commun Biol. 2023 Apr 18;6:428. doi: 10.1038/s42003-023-04808-z (PMC10113225; doi:10.1038/s42003-023-04808-z)
Supplement: Supplementary file 5 — nr-reporting-summary. [file 42003_2023_4808_MOESM5_ESM.pdf]

nature portfolio

Corresponding author(s): Dr Stéphane Mesnage

Last updated by author(s): Mar 29, 2023

Reporting Summary

Nature Portfolio wishes to improve the reproducibility of the work that we publish. This form provides structure for consistency and transparency in reporting. For further information on Nature Portfolio policies, see our Editorial Policies and the Editorial Policy Checklist.

Statistics

For all statistical analyses, confirm that the following items are present in the figure legend, table legend, main text, or Methods section.

n/a	Confirmed	
☐	☒	The exact sample size (*n*) for each experimental group/condition, given as a discrete number and unit of measurement
☐	☒	A statement on whether measurements were taken from distinct samples or whether the same sample was measured repeatedly
☐	☒	The statistical test(s) used AND whether they are one- or two-sided<br>*Only common tests should be described solely by name; describe more complex techniques in the Methods section.*
☐	☐	A description of all covariates tested
☐	☒	A description of any assumptions or corrections, such as tests of normality and adjustment for multiple comparisons
☐	☒	A full description of the statistical parameters including central tendency (e.g. means) or other basic estimates (e.g. regression coefficient) AND variation (e.g. standard deviation) or associated estimates of uncertainty (e.g. confidence intervals)
☐	☒	For null hypothesis testing, the test statistic (e.g. *F*, *t*, *r*) with confidence intervals, effect sizes, degrees of freedom and *P* value noted<br>*Give P values as exact values whenever suitable.*
☒	☐	For Bayesian analysis, information on the choice of priors and Markov chain Monte Carlo settings
☒	☐	For hierarchical and complex designs, identification of the appropriate level for tests and full reporting of outcomes
☒	☐	Estimates of effect sizes (e.g. Cohen's *d*, Pearson's *r*), indicating how they were calculated

Our web collection on statistics for biologists contains articles on many of the points above.

Software and code

Policy information about availability of computer code

Data collection	Microscopy data were collected on the manufacturer's proprietary software (NIS-elements). Data for Immunoblotting were collected using GeneSys (v1.6.7.0) and Biorad Chemidoc XRS+. The flow cytometry data were collected using the manufacturer's software (Guavasoft 3.2 and 3.4).
Data analysis	Statistical analyses were performed using Prism 9. For fluorescence microscopy and measuring features on the images, Fiji_1.53c and MicrobJ_5.13 were used.

For manuscripts utilizing custom algorithms or software that are central to the research but not yet described in published literature, software must be made available to editors and reviewers. We strongly encourage code deposition in a community repository (e.g. GitHub). See the Nature Portfolio guidelines for submitting code & software for further information.

Data

Policy information about availability of data

All manuscripts must include a data availability statement. This statement should provide the following information, where applicable:

- Accession codes, unique identifiers, or web links for publicly available datasets
- A description of any restrictions on data availability
- For clinical datasets or third party data, please ensure that the statement adheres to our policy

Raw data files are available on request. All data are kept on secure servers at the University of Sheffield for at least 10 years.

Human research participants

Policy information about studies involving human research participants and Sex and Gender in Research.

Reporting on sex and gender	N/A
Population characteristics	N/A
Recruitment	N/A
Ethics oversight	N/A

Note that full information on the approval of the study protocol must also be provided in the manuscript.

Field-specific reporting

Please select the one below that is the best fit for your research. If you are not sure, read the appropriate sections before making your selection.

☒ Life sciences ☐ Behavioural & social sciences ☐ Ecological, evolutionary & environmental sciences

For a reference copy of the document with all sections, see nature.com/documents/nr-reporting-summary-flat.pdf

Life sciences study design

All studies must disclose on these points even when the disclosure is negative.

Sample size	For microscopy experiments, no statistical methods were used to predetermine sample sizes. Sample sizes are given in the text and were chosen based on our prior experience and common standards in the field.
Data exclusions	N/A
Replication	All attempts at replication were successful
Randomization	N/A
Blinding	N/A

Reporting for specific materials, systems and methods

We require information from authors about some types of materials, experimental systems and methods used in many studies. Here, indicate whether each material, system or method listed is relevant to your study. If you are not sure if a list item applies to your research, read the appropriate section before selecting a response.

Materials & experimental systems

n/a	Involved in the study	
☐	☒	Antibodies
☒	☐	Eukaryotic cell lines
☒	☐	Palaeontology and archaeology
☒	☐	Animals and other organisms
☒	☐	Clinical data
☒	☐	Dual use research of concern

Methods

n/a	Involved in the study	
☒	☐	ChIP-seq
☐	☒	Flow cytometry
☒	☐	MRI-based neuroimaging

Antibodies

Antibodies used	Anti-AtlA antibodies were obtained from rabbits immunized with purified recombinant E. faecalis AtlA catalytic domain.<br>Anti-GFP antibodies were sourced from Sigma. Secondary antibodies used for Western blots are goat anti-rabbit IgG coupled to peroxidase (Sigma A0545). Secondary antibodies for immunofluorescence were sourced from ThermoFisher Scientific (RefA-21245)
Validation	Primary antibodies usqed for immunoblotting or immunofluorescence were validated using extracts or whole cells form E. faecalis wild-type and atlA null mutant (i-frame deletion)

Flow Cytometry

Plots

Confirm that:

- [x] The axis labels state the marker and fluorochrome used (e.g. CD4-FITC).
- [x] The axis scales are clearly visible. Include numbers along axes only for bottom left plot of group (a 'group' is an analysis of identical markers).
- [ ] All plots are contour plots with outliers or pseudocolor plots.
- [x] A numerical value for number of cells or percentage (with statistics) is provided.

Methodology

Sample preparation	Bacteria were grown overnight without agitation at 37C. Cells were diluted 1:100 in fresh growth medium and grown in standing cultures to mid-exponential phase (OD600nm=0.2-0.4. Cultures were diluter 1:100 in filtrated PBS before measurements.
Instrument	Cells were analysed by flow cytometry using a Millipore Guava easyCyte 6HT 2L system, serial number 8470060142.
Software	Guavasoft 3.2 and 3.4
Cell population abundance	N/A
Gating strategy	N/A

- [ ] Tick this box to confirm that a figure exemplifying the gating strategy is provided in the Supplementary Information.
